# Effectiveness of Microneedling With or Without Insulin in Patients With Acne: A Systematic Review and Meta-Analysis

**DOI:** 10.7759/cureus.104021

**Published:** 2026-02-21

**Authors:** Ali Aldoukhi, Merwi Alhadeyah, Fatemah Alshamari, Abdulrahman Makhseed, Ahmed Abdelaziz

**Affiliations:** 1 Department of Dermatology, Kuwait Institute for Medical Specialization, Kuwait City, KWT; 2 Department of Dermatology, Primary Healthcare Department, Ministry of Health, Kuwait City, KWT; 3 Department of Plastic Surgery, Sheikh Jaber Al-Ahmad Al-Sabah Hospital, Ministry of Health, Hawalli, KWT; 4 Department of Biostatistics, Faculty of Medicine, Al-Azhar University, Cairo, EGY

**Keywords:** acne vulgaris/therapy, adverse event, microneedling technique, systematic review and meta-analysis, topical insulin

## Abstract

Acne vulgaris is a common dermatologic condition that can lead to scarring, post-inflammatory hyperpigmentation, and psychosocial distress. Microneedling is an established minimally invasive therapy, and topical insulin has been proposed as an adjunct to enhance tissue repair and clinical outcomes. However, the comparative effectiveness of microneedling with or without topical insulin remains unclear. We performed a systematic review and meta-analysis of four trials including 259 patients to evaluate the efficacy and safety of microneedling combined with topical insulin versus microneedling alone. Outcomes assessed included categorical improvement (poor, moderate, good), mean improvement scores, and adverse events. Patients receiving microneedling plus topical insulin experienced a significantly lower rate of poor improvement compared with microneedling alone (risk ratio (RR): 0.35; 95% confidence interval (CI): 0.17-0.76; p = 0.01), while no notable observed differences for good improvement (RR: 2.96; 95% CI: 0.47-18.76; p = 0.25), moderate improvement (RR: 1.16; 95% CI: 0.68-1.98; p = 0.59), or mean improvement scores (standardized mean difference: 0.26; 95% CI: −0.07-0.58; p = 0.12). Adverse events were comparable between groups (RR: 1.53; 95% CI: 0.61-3.83; p = 0.36). Overall, microneedling remains an effective therapy for acne, and adjunctive topical insulin may reduce the risk of poor clinical response without increasing adverse events. These findings suggest potential benefit for selected patients, but larger, well-designed trials are needed to confirm efficacy and optimize treatment protocols.

## Introduction and background

Acne vulgaris is one of the most common dermatologic conditions worldwide, affecting adolescents and adults and often persisting beyond the teenage years [1]. Although frequently perceived as a benign disorder, acne can result in significant physical sequelae, including scarring and post-inflammatory hyperpigmentation, as well as substantial psychosocial distress [2]. The chronic and relapsing nature of acne, combined with variability in treatment response, continues to drive interest in procedural and adjunctive therapies that can improve clinical outcomes and patient satisfaction [3].

Microneedling has emerged as a minimally invasive treatment option for acne and acne-related scarring [4]. It creates tiny and micro-injuries in the human skin, which stimulates the production of collagen, enhances dermal remodelling, and improves transdermal drug delivery [4]. As a standalone modality, microneedling has demonstrated favorable effects on acne severity and skin texture with a relatively low risk profile. However, its ability to enhance the penetration of topical agents has prompted investigation into combination approaches aimed at optimizing therapeutic efficacy [5].

Topical insulin has gained attention as a potential adjunct to microneedling due to its biological effects on wound healing, keratinocyte migration, and fibroblast proliferation [6]. Insulin is known to promote tissue repair through the activation of growth factor pathways and enhancement of cellular regeneration. When applied topically following microneedling, insulin may augment the regenerative response and improve clinical outcomes in acne by accelerating healing and reducing inflammation [6]. Despite increasing clinical use of microneedling with topical insulin, the comparative effectiveness of this combination versus microneedling alone remains unclear.

Therefore, a systematic review and meta-analysis is warranted to pool the available data and clarify whether the addition of topical insulin provides a meaningful advantage over microneedling alone in patients with acne.

## Review

Methods

We carried out this systematic review and meta-analysis in accordance with the proposed criteria of the Preferred Reporting Items for Systematic Reviews and Meta-Analyses (PRISMA) [7] and followed the guidelines of the Cochrane Handbook for Systematic Reviews of Interventions [8].

Literature Search and Screening

A comprehensive literature search was performed using public databases including Scopus, PubMed, Web of Science (WoS), and Cochrane Central Register of Controlled Trials (CENTRAL) from inception to January 2026. The search strategy was terms related to acne, microneedling, and insulin. Key search terms included combinations of "acne vulgaris", "microneedling", "dermaroller", "dermapen", "topical insulin", and "insulin therapy". Detailed specific search strings according to each specific database are addressed in Table 1. Reference lists of relevant articles were manually screened to identify additional eligible studies. We did not apply any restrictions on publication status, while we included only English studies.

**Table 1:** Search strategy for each database. CENTRAL: Cochrane Central Register of Controlled Trials; WoS: Web of Science

Database	Search strategy	Results
PubMed	((("Acne Vulgaris/complications"[Mesh]) OR "Cicatrix"[Mesh] OR "Acne Vulgaris"[Mesh]) AND ("Collagen/administration and dosage"[Mesh] OR "Drug Delivery Systems"[Mesh]) AND ("Insulin/administration and dosage"[Mesh] OR "Insulin/therapeutic use"[Mesh])) OR (microneedle OR dermapen OR "skin needling") AND (insulin) AND (acne OR atrophic OR scar)	N=20
Scopus	TITLE-ABS-KEY((microneedling OR "micro-needling" OR dermapen OR "percutaneous collagen induction") AND (insulin) AND ("acne scar" OR "atrophic scar" OR "post-acne" OR "facial scar"))	N=15
WoS	((TI=(microneedling) OR TI=(dermapen)) AND TI=(insulin)) OR ((AB=(acne) AND AB=(scar)) AND AB=(insulin)) OR ((AK=(collagen induction) OR AK=(needling)) AND AK=(insulin))	N=13
CENTRAL	(microneedling or "micro-needling" or dermapen or "percutaneous collagen induction") AND (insulin) AND (acne and scar)	N=11

Eligibility Criteria and Endpoints

We considered studies if they met the following criteria: (1) involved patients diagnosed with acne vulgaris; (2) compared microneedling combined with topical insulin as the intervention group versus microneedling alone as the comparator; (3) reported quantitative clinical outcomes related to acne improvement; and (4) were clinical trials or observational cohort studies. Studies were excluded if they were review articles, conference abstracts without full text, or studies that did not clearly distinguish between the intervention and comparator arms.

Two independent reviewers removed duplicates and screened the studies judging titles and abstracts for relevance. Full-text articles were assessed for final inclusion if they met the inclusion criteria. Any diversities during the screening process were resolved through discussion until consensus was achieved.

The studied outcomes of this meta-analysis were the change in acne improvement scores from baseline to the end of follow-up, as reported in the included studies using validated or study-specific scoring systems [9], and categorical rates of clinical improvement, classified as poor, moderate, or good improvement according to each study's predefined criteria. Safety outcomes were also assessed, including the incidence of reported adverse effects such as erythema, pain, post-inflammatory hyperpigmentation, infection, or other treatment-related complications.

Quality Assessment

Two independent reviewers performed a methodological quality of the included studies using tools appropriate for each study design. Randomized controlled trials (RCTs) were evaluated using the Cochrane Risk of Bias (RoB 2) tool, which assesses bias across domains including random sequence generation, allocation concealment, blinding, incomplete outcome data, and selective reporting [10]. Non-randomized interventional clinical trials were assessed using the Risk of Bias in Non-randomized Studies of Interventions (ROBINS-I) tool [11]. This tool evaluates bias across seven domains, including bias due to confounding, selection of participants, classification of interventions, deviations from intended interventions, missing data, outcome measurement, and selective reporting. Observational cohort studies were assessed using the Newcastle-Ottawa Scale (NOS), which evaluates study quality based on the selection of participants, comparability of study groups, and outcome assessment [12]. Any discrepancies in quality assessment were resolved by consensus.

Data Extraction and Statistical Analysis

Two independent reviewers performed data extraction for the included studies using a standardized extraction form. Extracted data included study characteristics, sample size, patient demographics, acne severity at baseline, duration of follow-up, outcome measures, and reported adverse events.

Meta-analysis was performed when outcomes were reported by at least two studies with comparable measures. Continuous outcomes, including change in improvement scores, were pooled using standardized mean difference (SMD) with corresponding 95% confidence intervals (CIs). Categorical outcomes, including rates of poor, moderate, and good improvement as well as adverse events, were summarized using risk ratios (RRs) with 95% CIs. A random-effects model was applied to account for clinical and methodological heterogeneity across the included studies. Statistical heterogeneity was assessed using the I² statistic, with values greater than 50% indicating substantial heterogeneity. All statistical analyses were performed using the STATA 19 MP software (StataCorp LLC, College Station, TX, USA), and statistical significance was defined as a two-sided p-value of less than 0.05.

Results

Literature Review and Screening

**Figure 1:**
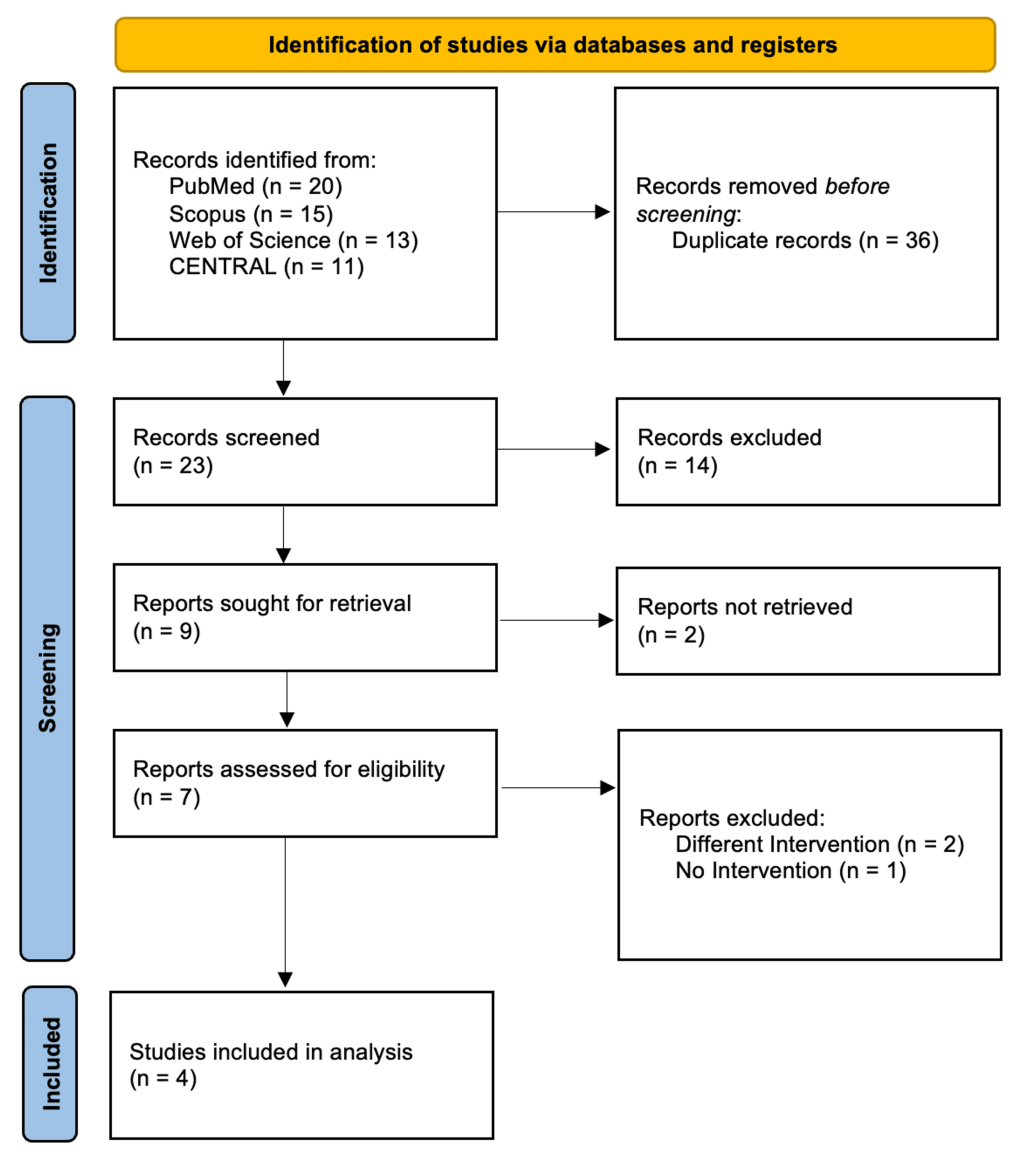
PRISMA flow diagram. PRISMA: Preferred Reporting Items for Systematic Reviews and Meta-Analyses

Our comprehensive literature search identified 59 records across all databases. After the removal of 36 duplicates, 23 articles remained for title and abstract screening. Fourteen articles were excluded at this stage, and two full texts could not be retrieved. The remaining seven articles underwent full-text assessment, of which four studies met the eligibility criteria and were included in the final quantitative analysis [13-16]. The study selection process is illustrated in the PRISMA flow diagram (Figure 1).

Characteristics and Quality Assessment

The included studies comprised mixed study designs. Two were split-face comparative clinical trials conducted in Pakistan [13,16], one was an RCT conducted in Egypt [14], and one was a prospective cohort study conducted in Syria [15]. Overall, the four studies included a total of 259 patients, of whom 184 (71%) were female. The weighted mean age of participants was 27.78 ± 6.88 years. In three studies, participants underwent both interventions, with microneedling combined with topical insulin applied to one side of the face and microneedling alone or placebo applied to the contralateral side. In contrast, the study by Karkour et al. [15] included parallel groups, with 119 patients in the intervention group and 39 patients in the control group. Detailed baseline characteristics of the included studies and participants are summarized in Table 2 and Table 3.

**Table 2:** Summary characteristics of the included studies. QoL: quality of life

Study	Country	Time frame	Study design	Patients, n			﻿Type of acne lesion	Inclusion criteria	Exclusion criteria	Study findings
				Total	Intervention	Control				
Ali et al. 2024 [13]	Pakistan	April-Sept 2023	Split-face comparative study	30	30	30	Atrophic post-acne scars (ice pick, boxcar, rolling, mixed): 60% ice pick, 20% boxcar, 13.4% rolling, 6.6% mixed	Post-acne atrophic scars, age 18-60, Fitzpatrick IV-VI	Active acne, diabetes, bleeding disorders, pregnancy/lactation	Insulin + ﻿microneedling showed superior improvement (grades II-III in 100% of patients) vs. ﻿microneedling alone (grades 0-I in 100%)
Abdelhay et al. 2025 [14]	Egypt	2022- 2023	Randomized controlled trial, split-face	21	21	21	Atrophic post-acne scars (types not specified in detail; likely ice pick, boxcar, rolling based on context)	Facial atrophic post-acne scars, ≥18 years	Active acne, infection, systemic disease, recent retinoid/laser, anticoagulants, keloid tendency, pregnancy/lactation	Both sides showed significant improvement, but no significant difference between insulin + ﻿microneedling and placebo + ﻿microneedling
Karkour et al. 2025 [15]	Syria	July 2021-Sept 2023	Prospective cohort	158	119	39	Atrophic scars from multiple causes: (1) post-acne scars (ice pick, boxcar, rolling), (2) post-leishmaniasis scars, (3) striae alba, (4) postoperative scars	Atrophic scars (acne, leishmaniasis, striae alba, postoperative)	Active infection, keloid history, diabetes, pregnancy, refusal	Significant improvement in acne and leishmaniasis scars with insulin + ﻿microneedling vs. placebo. Better QoL outcomes
Malik et al. 2024 [16]	Pakistan	Jan-July 2021	Prospective split-face comparative trial	50	50	50	Atrophic acne scars (ice pick, boxcar, rolling): study focused on atrophic types, but specific distribution not detailed	Atrophic acne scars, age 18-50, Goodman and Baron score ≥30	Active acne, bleeding disorders, keloid tendency, recent retinoid/laser, pregnancy/lactation	Both treatments were effective, but insulin + ﻿microneedling showed greater improvement and faster healing

**Table 3:** Baseline characteristics of the included patients. Data presented as n (%) and mean ± SD. NA: not assessed

Study	Group	Sample size	Age, years	Sex		Scar duration	﻿Fitzpatrick skin type		
				Male	Female		II	III	IV
Ali et al. 2024 [13]	Intervention	30	29.47 ± 8.36	12 (40)	18 (60)	2.03 ± 2.02	NA	NA	NA
	Control	30							
Abdelhay et al. 2025 [14]	Intervention	21	29.5 ± 9.0	5 (23.8)	16 (76.2)	10.5 ± 7.61	0 (0)	7 (33.3)	14 (66.7)
	Control	21							
Karkour et al. 2025 [15]	Intervention	119	26.2 ± 5.8	14 (12)	105 (88)	1.30 ± 1.2	NA	67 (56)	27 (23)
	Control	39	25.8 ± 6.2	15 (38)	24 (62)	2.25 ± 1.5	NA	28 (71)	10 (26)
Malik et al. 2024 [16]	Intervention	50	27.94 ± 5.06	29 (58)	21 (42)	NA	3 (6)	22 (44)	25 (50)
	Control	50							

Risk of bias assessment revealed overall some concerns to moderate risk of bias across the included studies. The RCT demonstrated some concerns, primarily related to limited reporting on allocation concealment and blinding procedures. The two non-randomized split-face clinical trials were judged to have a moderate risk of bias, mainly due to potential confounding and subjective outcome assessment. The prospective cohort study was assessed as having fair methodological quality, with limitations in comparability and outcome assessment.

Outcomes

**Figure 2:**
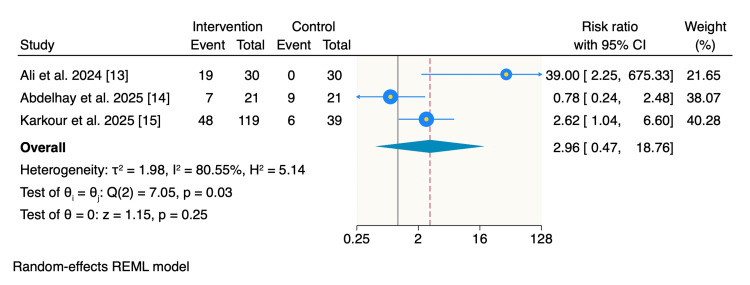
Random-effects model of rates of good improvement. REML: restricted maximum likelihood

Improvement categories: The proportion of patients reporting good improvement was higher in the intervention group with 43.5% (74 of 170 patients), compared with 16.7% (15 of 90 patients) in the control group. However, the pooled analysis did not demonstrate a statistically significant difference between groups (RR: 2.96; 95% CI: 0.47-18.76; p = 0.25), with substantial heterogeneity observed (I² = 80.55%) (Figure 2).

**Figure 3:**
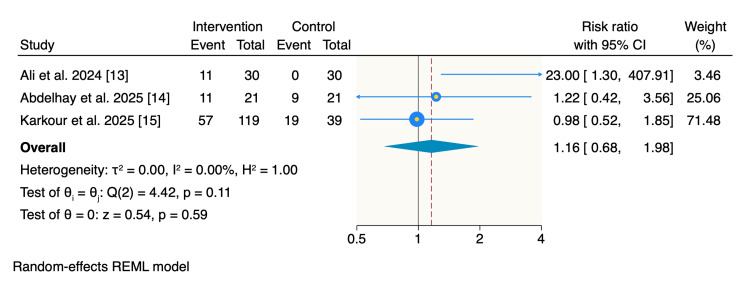
Random-effects model of rates of moderate improvement. REML: restricted maximum likelihood

Moderate improvement was reported by 79 patients (46.4%) in the intervention group and 28 patients (31.1%) in the control group. Meta-analysis showed no significant difference between the two groups (RR: 1.16; 95% CI: 0.68-1.98; p = 0.59), with no detected heterogeneity (I² = 0%) (Figure 3).

**Figure 4:**
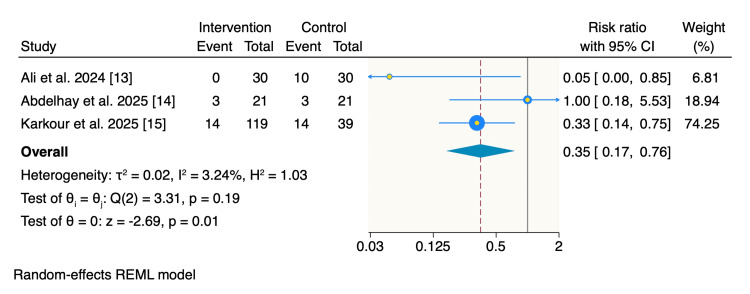
Random-effects model of rates of poor improvement. REML: restricted maximum likelihood

In contrast, poor improvement was reported significantly less frequently in the intervention group. Only 10% (17 of 170 patients) in the insulin group experienced poor improvement compared with 30% (27 of 90 patients) in the control group. The pooled estimate demonstrated a statistically significant reduction in poor improvement among patients receiving insulin (RR: 0.35; 95% CI: 0.17-0.76; p = 0.01), with minimal heterogeneity (I² = 3.24%) (Figure 4).

**Figure 5:**
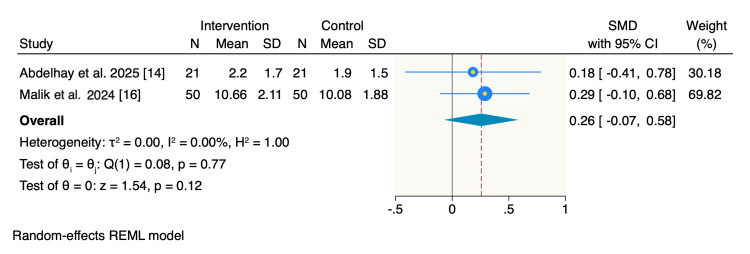
Random-effects model of mean change of improvement scores. REML: restricted maximum likelihood; SMD: standardized mean difference

Improvement scores: Improvement scores were calculated as the mean change between pre- and post-treatment assessments. Pooled analysis showed no statistically significant difference in improvement scores between the intervention and control groups (SMD: 0.26; 95% CI: −0.07 to 0.58; p = 0.12), with no heterogeneity observed (I² = 0%) (Figure 5).

**Figure 6:**
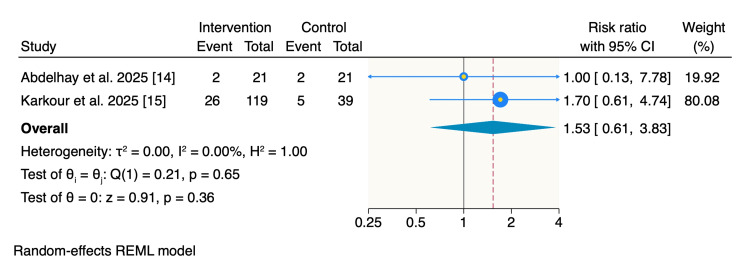
Random-effects model of rates of adverse events. REML: restricted maximum likelihood

Adverse events: Adverse events were reported in 20% of patients (28 of 140) in the intervention group compared with 11.7% (seven of 60) in the control group. However, the pooled analysis showed no statistically significant difference between the two groups (RR: 1.53; 95% CI: 0.61-3.83; p = 0.36), with no observed heterogeneity (I² = 0%) (Figure 6).

Discussion

This systematic review and meta-analysis evaluated the effectiveness and safety of microneedling combined with topical insulin compared with microneedling alone in patients with acne. By synthesizing evidence from four clinical studies, our findings suggest that the addition of topical insulin to microneedling may influence certain categorical clinical outcomes, particularly by reducing the proportion of patients experiencing poor improvement. However, these benefits did not consistently translate into statistically significant improvements across all measured outcomes, including overall improvement scores or rates of good and moderate response.

One of the most notable findings of this analysis was the significantly lower proportion of patients reporting poor improvement in the insulin-treated topics compared with microneedling alone. This suggests that topical insulin may contribute to more consistent clinical responses or help prevent suboptimal outcomes in a subset of patients [17,18]. In contrast, although higher proportions of patients in the insulin group achieved good improvement, this difference was not statistically significant and was accompanied by substantial heterogeneity. Similarly, no meaningful difference was observed between groups for moderate improvement or continuous improvement scores. Collectively, these findings indicate that while topical insulin may reduce treatment failure, its incremental benefit over microneedling alone remains uncertain in terms of achieving higher levels of clinical improvement [14,15].

The biological rationale for combining insulin with microneedling is well supported. Insulin is known to promote keratinocyte proliferation, fibroblast activation, collagen synthesis, and angiogenesis, all of which are essential for cutaneous repair and remodeling [19]. Microneedling enhances transdermal drug delivery by creating controlled microchannels in the epidermis, potentially improving insulin penetration and local bioavailability [20,21]. Together, these mechanisms may support accelerated healing and improved acne lesion resolution [22]. However, the lack of consistent improvement in standardized scores suggests that the magnitude of this effect may be modest or dependent on patient- or protocol-specific factors.

Importantly, no significant increase in adverse events was observed in patients receiving topical insulin. Although adverse events were numerically more frequent in the intervention group, the difference was not statistically significant, and reported events were mild and self-limited across studies. This supports the short-term safety of topical insulin when used in conjunction with microneedling, an important consideration for dermatologic therapies intended for cosmetic and quality-of-life improvement rather than life-threatening conditions [23].

Several methodological considerations may explain the variability in outcomes. The included studies differed in design, insulin concentration, microneedling devices, treatment frequency, follow-up duration, and outcome definitions [13-16]. Additionally, two studies employed split-face designs, which, while efficient, may introduce performance or assessment bias due to a lack of blinding [14,16]. The relatively small sample size and limited number of studies further reduce statistical power and increase susceptibility to imprecision, particularly for dichotomous outcomes with wide CIs.

From a clinical perspective, these findings suggest that microneedling combined with topical insulin may be a reasonable adjunctive approach for patients who exhibit suboptimal response to microneedling alone [15]. However, given the absence of consistent superiority across all outcomes, routine use of topical insulin cannot yet be recommended as standard practice. Instead, its use may be best reserved for selected patients within controlled or individualized treatment settings.

Our findings provide useful insights for dermatologists and clinicians managing patients with acne. Although the addition of topical insulin to microneedling did not significantly improve overall improvement scores or moderate-to-good outcomes, it was associated with a lower rate of poor response, suggesting potential benefit in selected patients [13]. Importantly, no significant increase in adverse events was observed, supporting the short-term safety of this combination therapy.

Future research should prioritize well-designed, powered RCTs with standardized outcome measures, longer follow-up durations, and consistent insulin dosing protocols. Particular attention should also be given to patient-reported outcomes, acne severity stratification, and objective scoring systems to better capture clinically meaningful benefits. Such studies are essential to clarify whether topical insulin offers a reproducible and clinically significant advantage beyond microneedling alone.

Limitations

This meta-analysis has several limitations. First, the number of included studies was small, and the overall sample size was limited, which reduced statistical power and contributed to imprecision in several pooled estimates. Second, heterogeneity in study design, including split-face trials, a single RCT, and an observational cohort, may have introduced variability in treatment effects and increased the risk of bias. Third, outcome definitions and improvement scoring systems were not fully standardized across studies, limiting direct comparability. Additionally, differences in insulin concentration, microneedling techniques, treatment frequency, and follow-up duration may have influenced the observed outcomes. Finally, the short-term nature of follow-up in most studies precludes the assessment of long-term efficacy and safety, including relapse rates and sustained improvement.

## Conclusions

In patients with acne, microneedling remains an effective therapeutic option, and the addition of topical insulin did not significantly improve overall or moderate-to-good improvement scores. However, insulin adjunct therapy was associated with a lower rate of poor response, without increasing adverse events, suggesting it may offer benefit for selected patients. Further well-designed trials with long-term follow-up are warranted to confirm these findings and optimize treatment protocols.

## References

[1] Vasam M, Korutla S, Bohara RA (2023). Acne vulgaris: a review of the pathophysiology, treatment, and recent nanotechnology based advances. Biochem Biophys Rep.

[2] França K, Keri J (2017). Psychosocial impact of acne and postinflammatory hyperpigmentation. An Bras Dermatol.

[3] Baldwin H, Frey C, Hebert A, Ted Lain E, Schlesinger T (2025). An algorithm integrating acneceuticals into the management of acne vulgaris. J Drugs Dermatol.

[4] Jaiswal S, Jawade S (2024). Microneedling in dermatology: a comprehensive review of applications, techniques, and outcomes. Cureus.

[5] Măgerușan ȘE, Hancu G, Rusu A (2024). Current understanding of microneedling procedures for acne skin: a narrative review. Cosmetics.

[6] Wang J, Xu J (2020). Effects of topical insulin on wound healing: a review of animal and human evidences. Diabetes Metab Syndr Obes.

[7] Page MJ, McKenzie JE, Bossuyt PM (2021). The PRISMA 2020 statement: an updated guideline for reporting systematic reviews. BMJ.

[8] Higgins JP, Thomas J, Chandler J, Cumpston M, Li T, Page MJ, Welch VA (2024). Cochrane Handbook for Systematic Reviews of Interventions. https://www.cochrane.org/authors/handbooks-and-manuals/handbook/current.

[9] Clark AK, Saric S, Sivamani RK (2018). Acne scars: how do we grade them?. Am J Clin Dermatol.

[10] Sterne JA, Savović J, Page MJ (2019). RoB 2: a revised tool for assessing risk of bias in randomised trials. BMJ.

[11] Sterne JA, Hernán MA, Reeves BC (2016). ROBINS-I: a tool for assessing risk of bias in non-randomised studies of interventions. BMJ.

[12] Stang A (2010). Critical evaluation of the Newcastle-Ottawa scale for the assessment of the quality of nonrandomized studies in meta-analyses. Eur J Epidemiol.

[13] Ali M, Ahmed N, Khan S, Kiran A (2024). Simple microneedling versus microneedling with topical insulin in the treatment of post acne atrophic scars; a split face comparative study. J Ayub Med Coll Abbottabad.

[14] Abdelhay RM, Ali MS, Gad LZ, Mahran NM (2025). Microneedling with topical insulin versus microneedling with placebo in the treatment of postacne atrophic scars: a randomized control trial. Dermatol Surg.

[15] Karkour B, Abu Ghedda S, Aljundi R, Sheikh Debs S, Farwati R, Ishkhanian S (2025). Efficacy and safety of microneedling with topical insulin compared with placebo in the treatment of atrophic scars: a prospective study and literature review. Skin Health Dis.

[16] Malik S, Tahir M, Ejaz Q, Kamal K, Kausar S, Sadaf S (2024). Comparison of micro needling plus topical insulin with micro needling alone in patients with atrophic acne scars: a split face study. Pak Armed Forces Med J.

[17] Sun S, Zhang L, Liu J, Li H (2021). Insulin topical application for wound healing in nondiabetic patients. Comput Math Methods Med.

[18] Sadowska-Przytocka A, Gruszczyńska M, Ostałowska A, Antosik P, Czarnecka-Operacz M, Adamski Z, Łącka K (2022). Insulin resistance in the course of acne - literature review. Postepy Dermatol Alergol.

[19] Liu Y, Petreaca M, Yao M, Martins-Green M (2009). Cell and molecular mechanisms of keratinocyte function stimulated by insulin during wound healing. BMC Cell Biol.

[20] Nguyen HX, Nguyen CN (2023). Microneedle-mediated transdermal delivery of biopharmaceuticals. Pharmaceutics.

[21] Zhao J, Xu G, Yao X, Zhou H, Lyu B, Pei S, Wen P (2022). Microneedle-based insulin transdermal delivery system: current status and translation challenges. Drug Deliv Transl Res.

[22] Nazary Abrbekoh F, Salimi L, Saghati S (2022). Application of microneedle patches for drug delivery; doorstep to novel therapies. J Tissue Eng.

[23] Hamed R, Abu Nahia BJ, Alkilani AZ, Al-Adhami Y, Obaidat R (2024). Recent advances in microneedling-assisted cosmetic applications. Cosmetics.

